# Clinical features of UK Biobank subjects carrying loss of function variants in genes implicated in schizophrenia pathogenesis

**DOI:** 10.1192/j.eurpsy.2022.306

**Published:** 2022-09-01

**Authors:** D. Curtis

**Affiliations:** University College London, Ucl Genetics Institute, London, United Kingdom

**Keywords:** loss of function variant, SETD1A, HERC1

## Abstract

**Introduction:**

The SCHEMA consortium has identified ten genes in which severely damaging variants substantially increase schizophrenia risk.

**Objectives:**

To characterise the clinical features of carriers of variants causing complete loss of function (LOF) of these genes.

**Methods:**

This research was conducted using the UK Biobank Resource and 200,000 exome-sequenced volunteers were screened to identify carriers of LOF variants in these genes. For these subjects, data fields were extracted which reflected educational and occupational functioning as well as clinical features including diagnoses and medication.

**Results:**

LOF variants in *CACNA1G* were commoner than in SCHEMA cases, suggesting this was not a real schizophrenia susceptibility gene. 159 subjects carried LOF variants in one of the other nine genes and overall they did not have poorer educational or occupational functioning or increased mental or physical health problems. Detailed examination revealed that one had schizophrenia, one had psychotic depression and two had a developmental disorder. Otherwise, a number of subjects had features of minor mental illness such as depression or anxiety and these rates were somewhat increased in subjects carrying LOF variants in *HERC1*, of whom more than half reported having consulted their GP for such problems. However the majority appeared to be entirely normal from a neuropsychiatric point of view.

**Conclusions:**

Although particular genetic variants can substantially increase the risk of schizophrenia, most people carrying them are entirely normal. This further supports the concept of schizophrenia as a distinct illness rather than representing the extreme of a trait which is present in the population.

**Disclosure:**

No significant relationships.

